# Comparison of non-subjective relative fungal biomass measurements to quantify the *Leptosphaeria maculans*—*Brassica napus* interaction

**DOI:** 10.1186/s13007-021-00822-6

**Published:** 2021-12-01

**Authors:** Wendelin Schnippenkoetter, Mohammad Hoque, Rebecca Maher, Angela Van de Wouw, Phillip Hands, Vivien Rolland, Luke Barrett, Susan Sprague

**Affiliations:** 1 CSIRO Agriculture and Food, 1 Clunies Ross Street, Canberra, ACT 2601 Australia; 2 School of BioSciences, The University of Melbourne, Parkville, VIC 3010 Australia

**Keywords:** Canola (*Brassica napus*), Blackleg (*Leptosphaeria maculans*), Disease resistance, Pathogen, Fungal biomass assay, Phenotyping

## Abstract

**Background:**

Blackleg disease, caused by the fungal pathogen *Leptosphaeria maculans*, is a serious threat to canola (*Brassica napus*) production worldwide. Quantitative resistance to this disease is a highly desirable trait but is difficult to precisely phenotype. Visual scores can be subjective and are prone to assessor bias. Methods to assess variation in quantitative resistance more accurately were developed based on quantifying *in planta* fungal biomass, including the Wheat Germ Agglutinin Chitin Assay (WAC), qPCR and ddPCR assays.

**Results:**

Disease assays were conducted by inoculating a range of canola cultivars with *L. maculans* isolates in glasshouse experiments and assessing fungal biomass in cotyledons, petioles and stem tissue harvested at different timepoints post-inoculation. PCR and WAC assay results were well correlated, repeatable across experiments and host tissues, and able to differentiate fungal biomass in different host-isolate treatments. In addition, the ddPCR assay was shown to differentiate between *L. maculans* isolates.

**Conclusions:**

The ddPCR assay is more sensitive in detecting pathogens and more adaptable to high-throughput methods by using robotic systems than the WAC assay. Overall, these methods proved accurate and non-subjective, providing alternatives to visual assessments to quantify the *L. maculans-B. napus* interaction in all plant tissues throughout the progression of the disease in seedlings and mature plants and have potential for fine-scale blackleg resistance phenotyping in canola.

**Supplementary Information:**

The online version contains supplementary material available at 10.1186/s13007-021-00822-6.

## Introduction

Precise and accurate quantification of disease severity is required to determine the genetic basis of resistance, breed cultivars resistant to disease, assess the efficacy of fungicides, and calculate relationships between crop losses and disease. While visual estimates of disease severity provide a relatively quick and easy phenotyping method, they can be subjective, difficult to reproduce and prone to error (Bock et al. [1]). Indeed, numerous studies have shown high levels of variability in assessments made by the same and different scorers (Kumar and Verma [2]). Assessment of pathogen biomass *in planta* provides an alternative to visual assessments and other image-based approaches to phenotyping disease resistance, promising potential for specific, rapid and objective quantification of quantitative disease resistance (Oliver et al. [3]).

Blackleg disease of canola is caused by the hemi-biotrophic fungal pathogen *Leptosphaeria maculans* (Howlett et al*.* [4]). Yield loss and plant death is typically associated with pathogen growth within the plant vascular system, resulting in reduced water and nutrient transport through the plant, and the development of crown canker. Crown cankers form after the fungus infects leaves, growing biotrophically through the petioles to the stem (Hammond et al. [5]). Plants are most susceptible to severe crown canker when seedlings are infected during early seedling growth (Marcroft et al. [6]; West et al. [7]).

Two forms of genetic resistance to *L. maculans* in canola have been identified: major gene resistance and quantitative resistance (Delourme et al*.* [8]). Major gene resistance (MGR) is conferred by race-specific single genes and is generally expressed qualitatively—either the host plant is resistant or susceptible. Quantitative resistance (QR) is thought to be a polygenic trait and is expressed as partial resistance (Raman et al. [9–12]; Delourme et al. [8]), in that it does not prevent infection but decreases canker severity. While both forms of resistance are useful, QR is desirable because it is considered race non-specific and appears to be more durable and so provide longer-term protection compared to MGR (Delourme et al. [13], Sprague et al*.* [14, 15], Brun et al. [16]). Most commonly, QR is assessed by visually scoring crown canker severity at maturity. Smaller leaf lesions and slower rate of hyphal extension along the petiole during the biotrophic phase (Huang et al. [17]) have been related to higher levels of QR, providing potential early screening methods for QR identification. The advantage of direct biomass quantification is that it provides non-subjective assessments with potential for high levels of accuracy and precision in the absence of visual symptoms. In addition, a non-subjective method could be used to differentiate *L. maculans* isolates for aggressiveness and to explore host–pathogen interactions.

Molecular and non-molecular methods have been previously developed for pathogen quantification in plant-fungal interactions. DNA-based quantitative polymerase chain reaction (qPCR) methods have been used to quantify several plant-fungus interactions (Klosterman [18]) and correlated with plant disease severity (Qi and Yang [19]; Brunner et al. [20]). Similarly, droplet digital polymerase chain reaction (ddPCR) can also be used to quantify pathogen DNA *in planta* but can provide absolute quantification of target nucleic acids without the need for calibration with known standards (Martinez-Diz [21], BIORAD Droplet Digital™ PCR Applications Guide [22]). Another non-subjective method for comparing fungal biomass in infected plant tissues has been developed by Ayliffe et al*.* [23] which describes a quantification assay relying on the strong binding affinity of the lectin Wheat Germ Agglutinin (WGA) to chitin, the prominent component of fungal cells walls. Wheat germ agglutinin (WGA) conjugated to the fluorophore fluorescein isothiocyanate (FITC), previously used for *in planta* visualisation of fungal infection structures at the microscope level (Ayliffe, et al. [24]), was used in a reportedly simple, sensitive, rapid and reliable histochemical assay called the WGA chitin assay (WAC).

The primary aim of this study was to compare different methods of assessing pathogen biomass and to determine their accuracy, precision, utility, and repeatability for phenotyping the *Leptosphaeria maculans-Brassica napus* interaction in different host tissues. In this study we compare the Wheat Germ Agglutinin (WGA) chitin assay or WAC (Ayliffe et al*.* [23]) and droplet digital PCR (ddPCR) assays for the detection and quantification of *L. maculans* fungal biomass in cotyledons, petioles and stems of canola throughout the disease progression.

## Materials and methods

Three complementary experiments were conducted in completely randomised glasshouse experiments (Table 1). Canola cultivars were selected to produce a range of disease severity, and included Westar, Pioneer Sturt, ATR-Stingray, ATR-Bonito, ATR-Mako, Hyola350TT and Bn1 (a non-commercial *B. napus* line). The *L. maculans* isolates used in the experiments (D2, D3, D8 and D22) were able to infect all cultivars except Hyola350TT which was used as a resistant control (Experiments 1 and 2 only). The cultivar Westar was used as a susceptible control as it is void of major resistance genes and has low levels of quantitative resistance. In Experiment 1, Hyola350TT was used as a resistant control as isolate D3 is avirulent (Navarrete [25], Van de Wouw, personal communication, Marcroft et al*.* [26]). Mock inoculations were performed for each experiment by inoculating complementary cultivar replicates with water as a control.

**Table 1:** Summary of experiments in this study

Experiment (Results)	Cultivars	*L. maculans* isolate	Tissue samples and sampling timepoints	Biological replicates	Location and time of year
**1** (Fig. 1 and Additional file 1: Fig. S1)	Westar, Pioneer Sturt TT, ATR-Stingray, ATR-Mako, ATR-Bonito, Hyola®350TT	D3	Cotyledons 12 dpi	10 (ddPCR) 3 (WAC)	Canberra, ACT Nov-Dec 2019
**2** (Figs. 2, 3 and 4)	Westar, Bn1, ATR-Mako, ATR-Bonito, Hyola®350TT	D2	Cotyledons and petioles 9 dpi (T1) 12 dpi (T2) Crowns and hypocotyls 4 wpi (T3) 7 wpi (T4) 12 wpi (T5)	14	Horsham, Victoria Mar-Jun 2020
**3** (Fig. 5)	Westar ATR-Bonito ATR-Mako	D2 D8 D22	Cotyledons 12 dpi	40	Canberra, ACT Aug 2020

Canola cultivars, *L. maculans* isolates, tissue types, sampling timepoints and replication for three glasshouse experiments. *Dpi*  days post-inoculation and *wpi * weeks post-inoculation

**Fig. 1:**
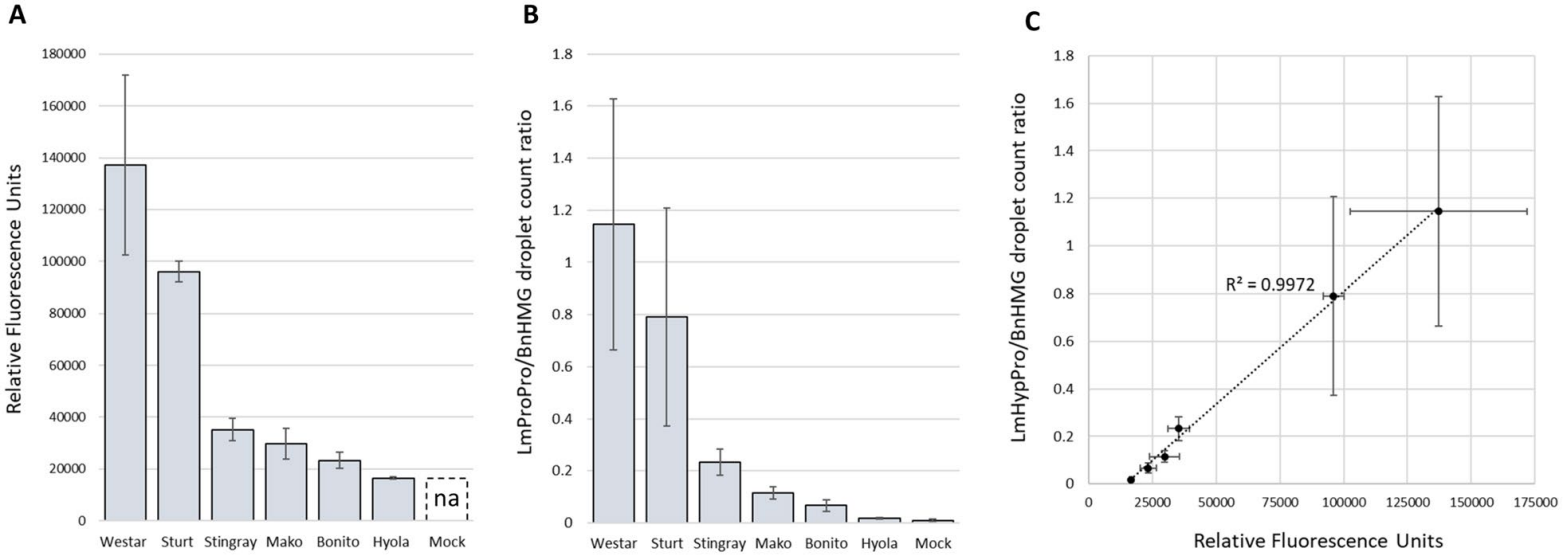
*L. maculans* fungal biomass assays of 12 dpi cotyledons (Experiment 1). *L. maculans* pathogen load measured using the WAC assay **A** and ddPCR **B** at 12 dpi in cotyledons of six *B. napus* cultivars inoculated with isolate D3. Mocks were not processed in the WAC analysis (**A**, *na* not available). **C** Linear regression analysis set at 95% confidence level between the WAC and ddPCR fungal biomass assays revealed an adjusted R^2^ value of 0.9965 indicating a strong correlation between the results obtained from the WAC and ddPCR fungal biomass assay measurements. Standard error bars included in the regression and bar diagrams represent 3 biological replicates for the WAC assay (**A**) (P < 0.0005) and 10 for the ddPCR **B** (P < 0.003). Sturt, Stingray, Mako, Bonito and Hyola refer to cvs Pioneer Sturt TT, ATR-Stingray, ATR-Mako, ATR-Bonito and Hyola350TT respectively

**Fig. 2:**
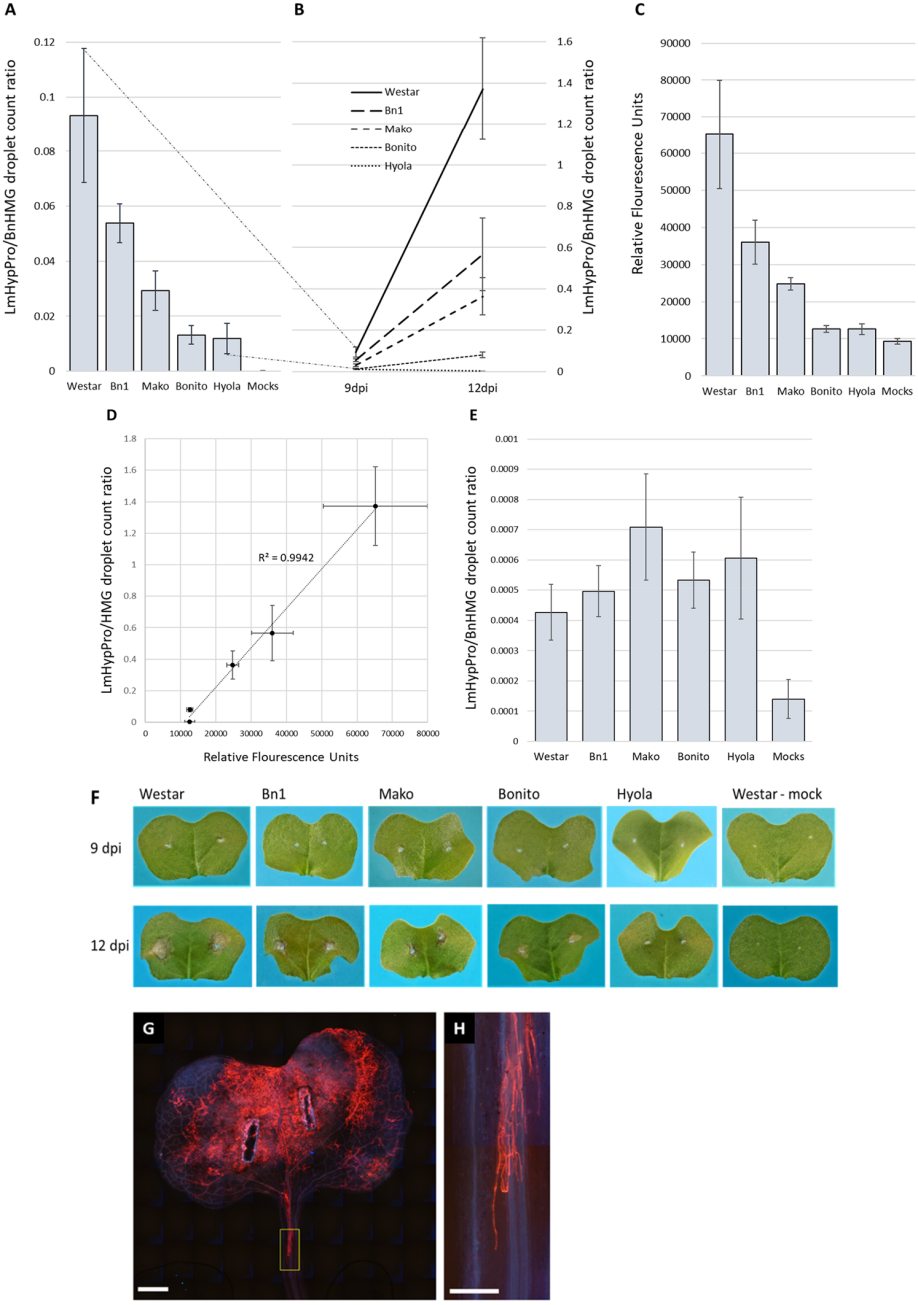
*L. maculans* fungal biomass assays of, and progression in, 9 and 12 dpi cotyledons (Experiment 2).** A** ddPCR tests on DNA extracted from D2 infected cotyledons 9 dpi from canola cultivars in this study (P < 5 × 10^–7^). **B** Disease progression ddPCR results between 9 and 12 dpi timepoints (P < 1.5 × 10^–8^ at 12 dpi). Bar diagram **A** distinguishes cultivars Bn1, ATR-Mako and ATR-Bonito (referred to as Mako and Bonito respectively) from each other and all from susceptible Westar at 9 dpi—broken lines extending from diagrams **A**, **B** indicate the breadth of scale in the progression shown in **B**. (Hyola350TT is referred to as Hyola). Standard error bars represent 14 biological replicates. **C**: Wheat germ agglutinin (WGA) chitin assay (WAC) performed on the respective 2nd cotyledons of the 12 dpi timepoint samples used for ddPCR (**B**). Relative fungal biomass assays indicate similar trends between 1st cotyledons subjected to ddPCR assays **A**, **B** and the WAC assay **C** respectively. Standard errors included in bar diagrams and progression diagram represent the mean from 14 biological replicates (P < 8 × 10^–8^). (ATR-Mako, ATR-Bonito, and Hyola®350TT cultivars are referred to as Mako, Bonito and Hyola, respectively). **D** Linear Regression test on the respective 12 dpi cotyledon WAC and ddPCR data set at confidence level of 95% obtained an adjusted R^2^ value of 0.9942 indicating a strong correlation between the results of the WAC and the ddPCR. Standard error bars for X- and Y-axes are shown for each mean data point, respectively. SE bars represent 14 biological replicates. **E** ddPCR results on the DNA extracted from the petiole samples removed from infected cotyledons which were sampled 9 dpi (data for 12 dpi not shown). Standard error bars represent 14 biological replicates (P < 0.061). (ATR-Mako, ATR-Bonito, and Hyola350TT cultivars are referred to as Mako, Bonito and Hyola, respectively). **F** Representative infected cotyledons sampled at 9 and 12 dpi respectively. **G** and **H**: Epifluoresence image of a WGA-TRITC stained ATR-Stingray cotyledon at 14 dpi. *L.maculans* hyphae spread (orange) from the inoculation points (**G**) and an enlargement of the yellow boxed region (**H**) showing hyphal growth in cells surrounding the vascular strand (blue) in petiole. Scale bars show 5 and 2 mm in G and H, respectively

**Fig. 3:**
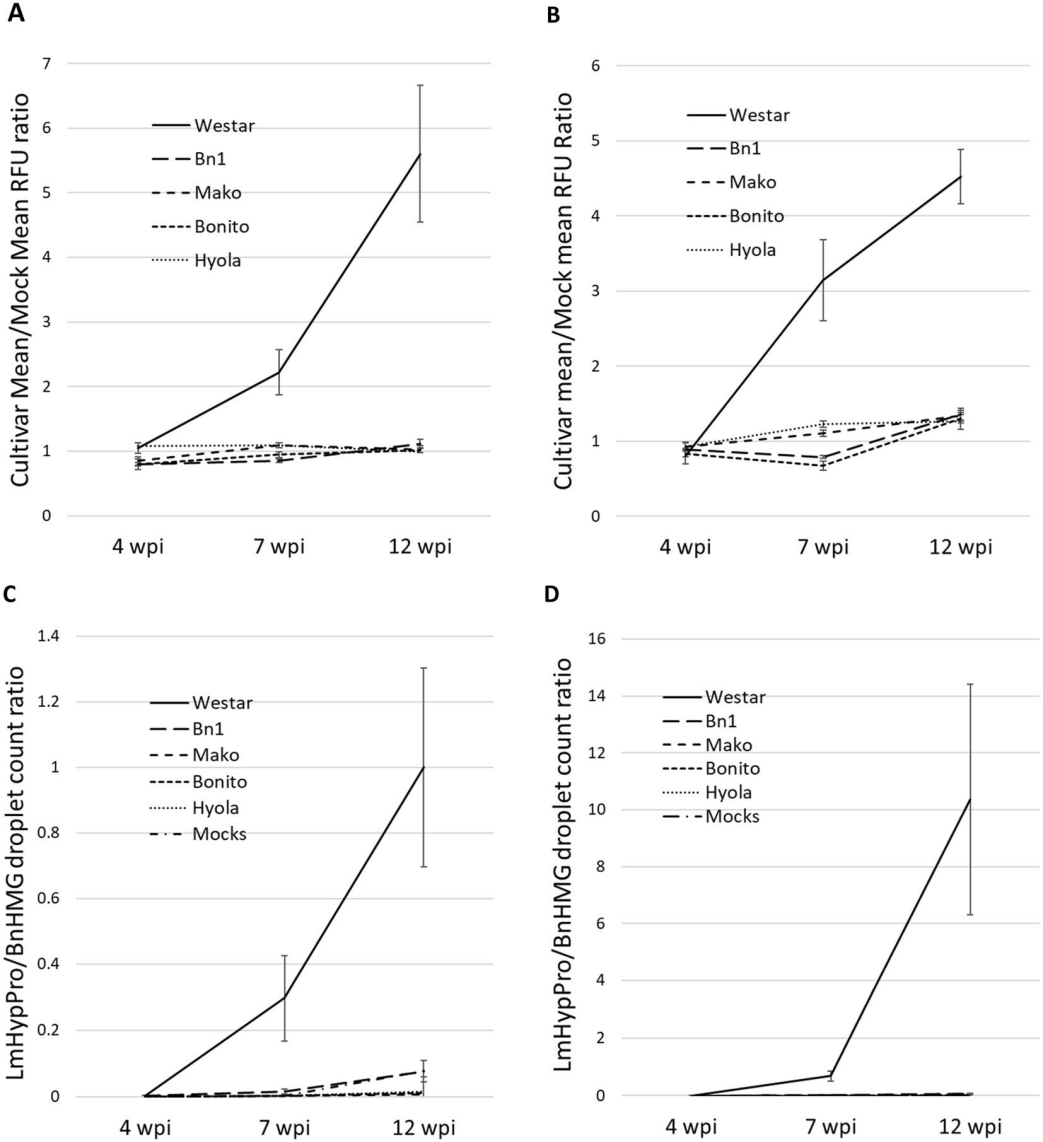
*L. maculans* fungal biomass assays of, and progression, in stems (Experiment 2). WAC assays (**A**, **B)** and ddPCR assays (**C**, **D)** of the crown (**A**, **C)** at the cotyledon petiole-stem junction and hypocotyl (**B**, **D)** directly below the crown. WAC and ddPCR results revealed similar trends in WAC and ddPCR for both crown (**A,** **C)** and the hypocotyl sections (**B**, **D**). WAC (diagrams **A**, **B**) y-axes scales are presented as ratios of cultivar RFU means/mock background RFU means. Standard error bars in WAC assays were calculated based on at least three technical replicates for each of 14 biological replicates. Standard error bars in ddPCR assays (**C**, **D)** were calculated based on 14 biological replicates. (ATR-Mako, ATR-Bonito, and Hyola350TT cultivars are referred to as Mako, Bonito and Hyola, respectively). P < 0.02 for all timepoint data sets except for 4 wpi (T3) ddPCR data set **C** where P < 0.16

**Fig. 4:**
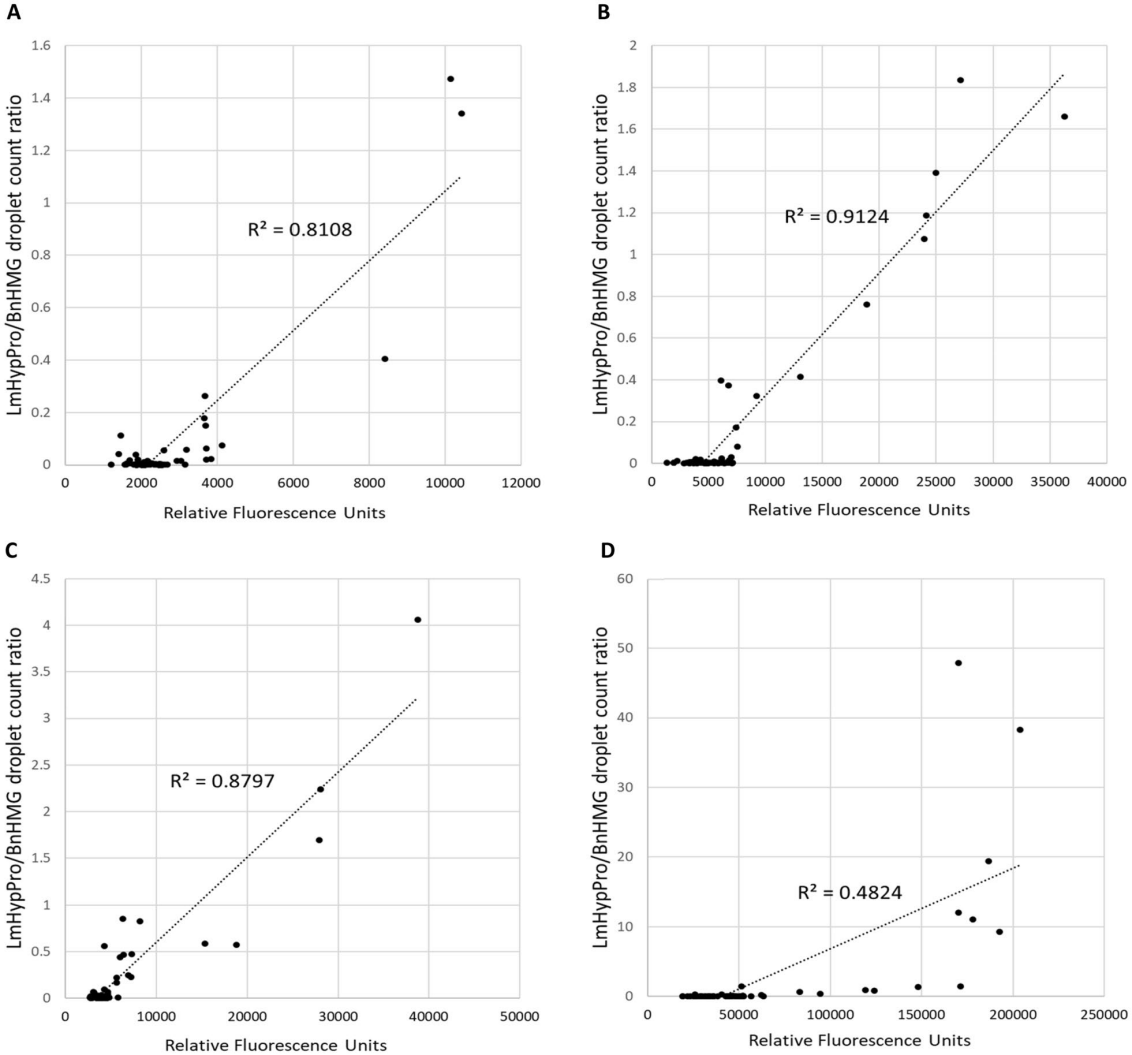
Linear regression tests on crown and hypocotyl assay data (Experiment 2). Linear regression analyses on individual WAC versus ddPCR data points revealed a correlation with R^2^ values of 0.8108 for 7 wpi stem crowns (**A**), 0.9124 for 7 wpi hypocotyls (**B**), 0.8797 for 12 wpi stem crowns (C) and 0.4824 for 12 wpi hypocotyls (**D**). Adjusted R^2^ values are 0.8079, 0.9111, 0.8779 and 0.4741, respectively. PCR and WAC assay results correlated for 7 wpi crown and hypocotyl data as well as for 12 wpi crown data however this correlation fell away for 12 wpi hypocotyl sample data at the latter stages of the infection life cycle of *L.maculans*

**Fig. 5:**
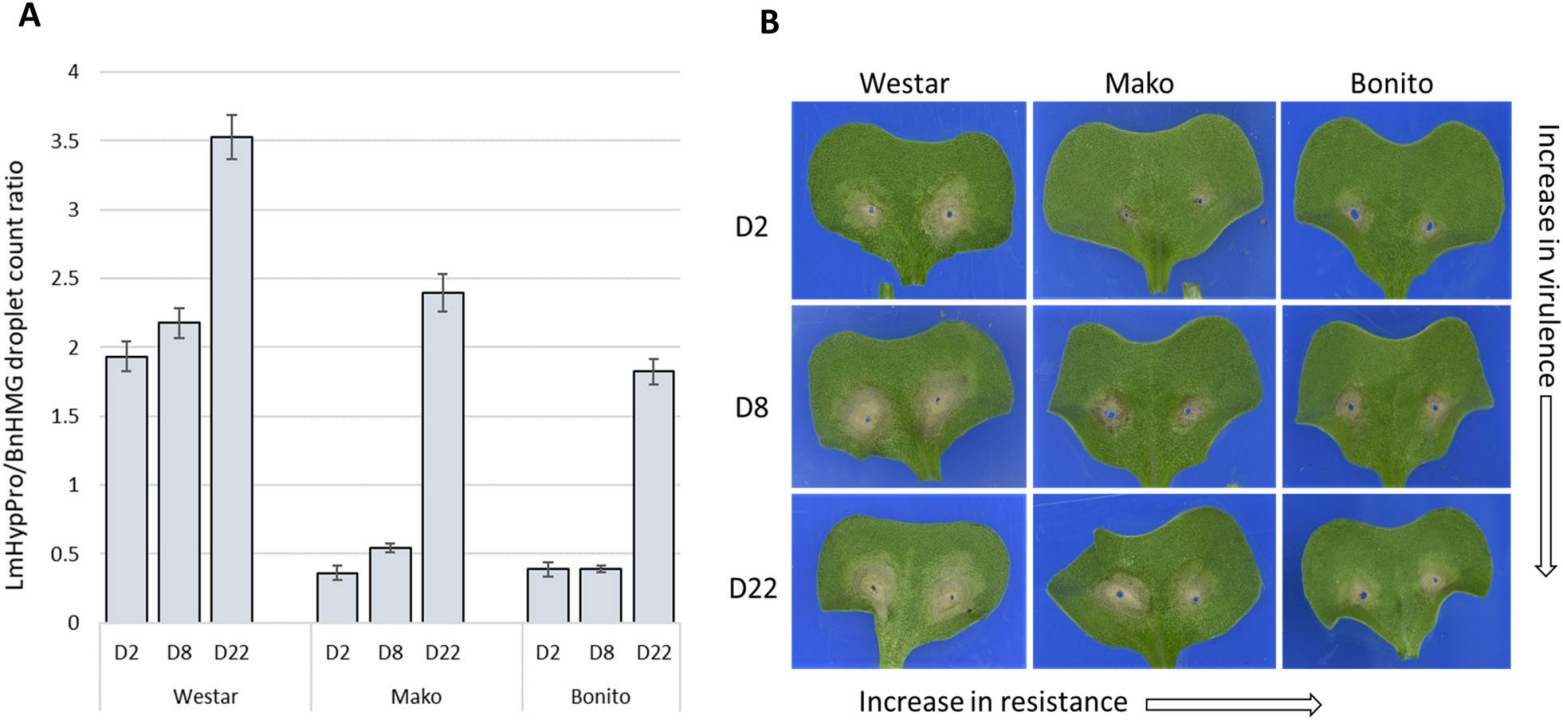
*L. maculans* isolate ddPCR assay comparisons of *B. napus* cultivar cotyledons (Experiment 3).** A** *L. maculans* biomass measured by ddPCR in three *B. napus* cultivars infected with isolates D2, D8 and D22. Cotyledon samples were harvested at 12 dpi. Standard error bars represent 40 biological replicates for all isolate or cultivar comparisons (P < 1.7 × 10^–15^). **B** Representative cotyledon samples for the respective isolates and cultivars arranged in order of cultivar resistance and pathogen virulence. (ATR-Mako and ATR-Bonito cultivars are referred to as Mako and Bonito, respectively)

The aim of Experiment 1 was to compare WAC and PCR techniques for measuring the relative fungal biomass in cotyledons inoculated with the *L. maculans* D3 isolate, using 10 replicate plants per isolate x cultivar treatment (Fig. 1 and Additional file 1: Fig. S1). Cotyledons (without their petioles) were harvested at 12 dpi prior to the senescence of cotyledons and processed for WAC (3 biological replicates) and PCR analyses (10 biological replicates).

The aims of Experiment 2 were to (i) assess assay repeatability under different environmental conditions and using a broader set of cultivars inoculated with a different isolate, and (ii) determine whether the assays could quantify blackleg infection as disease progressed from cotyledon lesions, through the petiole and into the stem (Figs. 2, 3 and 4). Cotyledons of five canola cultivars were inoculated with *L. maculans* isolate D2, using 14 replicate plants for each cultivar at each of the five assessment timepoints (Figs. 2, 3 and 4). At 9 and 12 dpi, the pairs of cotyledons (lobes and petioles) from each plant were detached from the plant at the stem. Petioles were separated from their cotyledons to enable blackleg quantification in each tissue type. Stem sections were sampled at 4, 7 and 12 weeks post-inoculation (wpi), coinciding approximately with the start of flowering, end of flowering and physiological maturity, respectively. For each plant, two 1 cm sections of stem were sampled using secateurs: one immediately above and one immediately below the cotyledon petiole scar at the crown. Fungal biomass was quantified separately for the two tissue sections.

The aim of Experiment 3 was to determine whether the ddPCR assay could be used to phenotype the aggressiveness of *L. maculans* on cotyledons (Fig. 5). Cotyledons of three cultivars were inoculated with three *L. maculans* isolates with cotyledons comprising the lobes (but not the petioles) harvested at 12 dpi, using 40 replicate plants for each cultivar x isolate treatment.

### Canola growth and inoculation

*Leptosphaeria maculans* isolates were cultured on V8 agar plates (16% V8 juice in 1% agar preparation) in a fluorescence lamp-lit 20 °C incubator with a 12 h light/dark cycle. D8 was observed to grow slower than D2 or D22 on V8 agar plates (Van de Wouw, personal communication). Pycnidiospores were harvested at 10–14 days with suspensions filtered through sterile cheesecloth, quantified using a haemocytometer and stored at − 20 °C. Prior to inoculation, aliquots were thawed, and the concentration adjusted to 1 × 10^6^ spores/mL with sterile water containing Tween20 surfactant (1–2 drops/250 mL).

Seeds were sown in square pots (50 × 50 × 120 mm) in the glasshouse and inoculated when the cotyledons had unfolded (7–9 days post-sowing). The lobe of each cotyledon was wounded (4 per plant) by pin-pricking a hole 2–3 mm from the junction of the lobe and petiole and 2–3 mm from the central cotyledon venation. A 10 µL drop of inoculum (≥ 1 × 10^6^ pycnidiospores/mL with Tween20 surfactant) was pipetted onto each wound. Developing true leaves were removed once at 4 dpi to delay cotyledon senescence and allow for the progression of the infections through the petiole to the stem.

### Plant tissue preparation and DNA extractions

For Experiments 1 and 2, cotyledon, petiole, crown and hypocotyl were sampled into envelopes or 2 mL Eppendorf tubes (with the tube tops punctured). For Experiment 3, whole cotyledons were placed into 96-deep well plates at the time of sampling. All samples were frozen at − 80 °C for at least 2 h prior to freeze-drying. Freeze-dried cotyledons and petioles in either 2 mL Eppendorf tubes or plates were ground with a single 5 mm steel ball-bearing using a Qiagen® TissueLyser II™ set at a frequency of 20–22 Hz for 2 min. Freeze-dried stem samples were ground separately using the CT 293 Cyclotec™ Labtec™ Line laboratory mill (FOSS®) containing a 2 mm perforated screen with 0.05 g portions transferred to either 2 mL Eppendorf tubes for chitin assay analysis or 96-well plates for high-throughput genomic DNA extractions.

For Experiments 1 and 2, gDNA from whole freeze-dried cotyledons was extracted in 2 mL Eppendorf tubes using Nucleospin® Plant II DNA Purification Kit (Macherey–Nagel; www.mn-net.com) and quantified at 260 nm wavelength absorbance on a NanoDrop® 2000c spectrophotomer (NanoDrop Technologies, Wilmington, DE, USA).

For Experiment 3, gDNA extractions were performed according to methods described in Ellis et al. [27] with modifications for transfers using a Hamilton™ Nimbus Robot. Ground material was freeze-dried in 96-well microtiter plates (Integrated Sciences). The ground material was incubated for 1 h at 65 °C with 375 µL extraction buffer (0.1 M Tris–HCl pH 8, 0.05 M EDTA pH 8, 1.25% SDS, 0.3 µg/mL RNase A). 187 µL 6 M ammonium acetate was added to the samples, which were then incubated for 15 min at 4 °C and centrifuged (30 min at 1,610 g). The supernatants were then precipitated (220 µL isopropanol, 5 min room temperature, 30 min centrifugation at 1,610 g). The pellets washed with 70% ethanol, briefly airdried and dissolved in 225 µL distilled water overnight. The plates were then centrifuged (1,610 g) and 150 µL of the extracts transferred to a clean 96-well plate.

### WGA-FITC chitin assay (WAC)

The methods described by Ayliffe et al*. *[23] for comparing relative fungal biomass in infected plant tissues in the form of WAC assays were modified for *L. maculans* in canola. Approximately 0.05 g portioned ground material was suspended in 500 µL 1 M KOH, incubated at 65 °C for 2–4 days and subjected to mixing at least twice daily. Suspensions were centrifuged at 2000 × *g* for 3 min and supernatants discarded. Pellets were washed 3 or 4 times by resuspending in 300–500 µL 50 mM Tris–HCl pH 7.5 buffer and centrifuging at 2000 × *g* for 3 min and discarding the supernatants. The optical densities of the final suspensions were adjusted to 1.0 at 600 nm wavelength to ensure the amount of plant material being measured was standardised.

Three to four 200 µL technical replicates of each final suspension of each sample were mixed in 5 µL 1 µg/mL WGA-FITC conjugate stain (Lectin from *Triticum vulgaris* (wheat) FITC conjugate, lyophilised powder from SIGMA® (L4895) in PCR tube strips. Samples were incubated at room temperature for at least 1 h then centrifuged at 2000 × *g* for 3 min and supernatants discarded. To remove excess WGA-FITC stain, pellets were washed 3 or 4 times as described above. Each replicate pellet was resuspended in 150 µL Tris–HCl pH 7.5 buffer and transferred to a well in a black non-transparent 96-well plate. Relative fungal biomass measurements at 485 nm absorbance and 520 nm emissions were determined as a relative measure using the Omega FLUOstar™ Microplate Reader and its Software version 5.11 R4 from BMG Labtech. Omega Data Analysis Software MARS was used to transfer the readings to Microsoft Excel for analysis.

### PCR Primer and ddPCR probe design

Primers and probes used to detect the respective single-copy reference genes (Table 2) were designed using Primer3Plus software available on http://primer3plus.com/ (Rozen and Skaletsky [28]). *L. maculans* Hypothetical Protein specific primers (LmHypPro-F and -R) and probe were used for species identification was designed using the Geneious Prime™ Software. *Leptosphaeria maculans* primer PCR products were subjected to sequencing and a NCBI BLAST analysis indicated specificity to this pathogen only. Candidate primers were adjusted to have annealing temperatures of approximately 58 °C while fluorescently labelled probes for amplicon detection were selected to have annealing temperatures of approximately 66 °C. Candidate primers, respective primer pairs and probes were tested for the formation of primer dimers or multiplex primer dimers or hairpin loops (at a < − 2.0 ΔG threshold) at the annealing temperature of 58 °C using Primer3Plus software. Amplicon sequences were tested for folding on AutoDimer Software (https://strbase.nist.gov//AutoDimerHomepage/AutoDimerProgramHomepage.htm) (at a < − 2.0 ΔG threshold) so the primer binding sites remained open and devoid of hairpins and the probe binding site remained in open configuration in PCR reactions. *Brassica napus* reference primer sequences were obtained from Petrie et al*.* [29] (Table 2). Previously, qPCR has been used to quantify *L. maculans* fungal biomass using *L. maculans*-specific primers (Huang et al. [30, 17, 31]; Liu et al. [32]; Voigt et al. [33]). Primers LmacF and LmacR, specific for amplification of the internal transcribed spacer (ITS) region of ribosomal DNA or actin of *L. maculans* (Voigt et al*.* [33]), that have been previously used for identification (Liu et al. [32]) and quantification (Huang et al. [30]) of *L. maculans* were not found to be entirely specific to *L. maculans* in BLAST NCBI analyses.

**Table 2:** Primer pairs obtained from SIGMA were tested by conventional endpoint PCR to confirm a single product of the correct size was amplified

**Reference gene** **Sequence and source**	**Primer name**	**Sequence (5’-3’) and melting temperature (Tm)**	**Amplicon**
*Leptosphaeria maculans* Hypothetical Protein (LEMA_P025150.1)	LmHypPro-F	GCGCGAATCACCAGATACA (Tm = 58.0 °C)	124 bp
	LmHypPro-R	CTCCTCTAGGGAAGGACATACA (Tm = 57.8 °C)	
	LmHypPro-Pr	5’-/56-FAM/TGCCGCGCT/ZEN/GGTATAGTCCGAT/3IABkFQ/-3’ (Tm = 66.1 °C)	
*Brassica napus* High Mobility Group Protein (NM_001316094.1) (Petrie et al*.* [29]; Weng et al. [34, 35])	BnHMG-F	GCGAAGCACATCGAGTCA (Tm = 57.8 °C)	73 bp
	BnHMG-R	GGTTGAGGTGGTAGCTGAGG (Tm = 59.7 °C)	
	BnHMG-Pr	5’-/5HEX/TCTCTACCA/ZEN/CCGTCTCACATGACGC/3IABkFQ/-3’ (Tm = 66.0 °C)	

The two reference gene probes were obtained from IDT Integrated Technologies® which were labelled with 5’ FAM™ (6-flourescein) and 5’ HEX™ (hexachloro-flourescein) respectively and both were double-quenched with ZEN™ and Iowa Black Hole Quencher®

### ddPCR quantification of L. maculans biomass in infected cotyledons

For each sample, a 1 µg gDNA sample was double digested with restriction endonucleases EcoR1 and BamH1 in a volume of 100 µL at 37 °C overnight. A 3 µL sample of the digested gDNA containing approximately 300 ng fractionated DNA was added to a volume of 22 µL ddPCR master-mix comprising 12.5 µL BIO-RAD® 2xddPCR™ Supermix for Probes (no dUTP), 1 µL of 10 µM of each of the 4 primers listed in Table 2, 0.5 µL 10 µM of each of the 2 probes listed in Table 2 and 2.5 µL MQ® water. Droplets were generated from 20 µL of each of the complete reaction mixtures drawn together with 70 µL BIO-RAD® Droplet Generation Oil respectively, in the microcapillary droplet generator cartridge following manufacturer’s instructions (Instruction Manuals; BIO-RAD® QX100™ Droplet Generator™ or the Automated Droplet Generator™; BIO-RAD® Droplet Digital™ PCR Applications Guide [22]). A 40 µL sample of each emulsified droplet preparation was carefully transferred from the droplet generation cassette to the 96-well PCR plates according to manufacturer’s instructions with a Rainin 50 µL eight-channel multi-pipettor. Plates were heat sealed with pierceable foil using the BIO-RAD® PX1 PCR plate sealer and placed in a BIO-RAD® Touch C1000 thermocycler. The conditions for amplification consisted of an initial 95 °C denaturisation for 10 min, followed by 40 cycles of 94 °C for 30 secs and 60 °C for 1 min. A temperature ramp rate of 2.5 °C sec^−1^ was set for all temperature changes. A final 10 min 98 °C step followed the 40 cycles. Samples were transferred to a BIO-RAD® QX200 or QX100 droplet Reader. Droplet counts, analyses and droplet pathogen/host number measurements were generated using the BIO-RAD® QuantaSoft™ software with the default settings for threshold determination to distinguish positive and negative droplets (Petrie et al. [29]). The ddPCR analyses did not require technical replicates since repeat experiments of at least 14 DNA samples indicated near identical results with a regression test revealing a R^2^ = 0.9956 value (Additional file 1: Fig. S1).

On selected DNA samples used in ddPCR tests, qPCR was performed to ensure the ddPCR results were accurate and consistent. Approximately 50 ng of each of the selected samples was used as a template for qPCR analysis. qPCR reactions were performed using iTaq Universal SYBR Green Supermix and the CFX96 Touch Real-Time PCR Detection System from BIO-RAD™. Reaction conditions included an initial denaturization at 95 °C for 3 min; 40 cycles of denaturization at 95 °C for 10 s and annealing/elongation at 58 °C for 30 s, followed by a melt step range of 65–95 °C with increments of 0.5 °C. A 124 bp product of *L. maculans* hypothetical protein DNA sequence was amplified from gDNA using the LmHypPro-F and LmHypPro-R primers while a 73 bp product of *B.napus* HMG-Y-related protein DNA sequence was amplified using the BnHMG-F and BnHMG-R (Table 2). The amount of PCR product generated by each primer pair was quantified by absolute quantification method of comparison (Brankatschk et al*.* [36]) with respective standard curves using Maestro BIO-RAD™ Software. The ratio of product produced by *L. maculans* hypothetical protein PCR product and *B. napus* HMG-Y-related protein PCR product was taken as a measure of relative fungal biomass. Each experiment had at least three technical replicates for each plant sample (biological replicate) and was repeated by using the ddPCR technique described below (Boulter et al*.* [37]).

### Microscopy

Cotyledons including the petiole were collected from plants at various time points following inoculation. Inoculated cotyledons were immersed in farmers fixative (3:1 Ethanol: Acetic Acid). Fixative was replaced after 24 h and again at least 1 h before staining to remove remaining chlorophyl. Samples were stored in fixative at room temperature. Tissue clearing and staining followed the method described in Ayliffe et al*.* [24]. Samples were cleared with 10% (w/v) potassium hydroxide (KOH) by heating at 85 °C for approximately 8 min. KOH/heat treatment was stopped when samples had optical clarity to maintain structural integrity. KOH solution was discarded, and samples were subjected to four times 10-min PBS (Phosphate-Buffered Saline, pH 7.4) washes. Samples were stained in PBS containing 20 µg/mL WGA-TRITC conjugate (Thermo Fisher Scientific, MA, USA) at room temperature for 30 min with occasional gentle agitation. Staining solution was discarded, and samples were washed twice in PBS, pH 7.4 for 2 min before mounting in PBS on glass slides. Widefield images were obtained using a Zeiss AxioImager Z1 microscope equipped with LED illumination (Colibri 7), Zeiss Axiocam 712 colour CCD camera (Carl Zeiss Micro-imaging GmbH, Jena, Germany) and using a plan-apochromat 10x/0.3 objective. Zeiss filter sets 43 HE (excitation BP550/25; beam splitter FT 570 HE; emission BP605/70 HE) and 02 (excitation 365; beam splitter 395; emission LP 420) filters were used to visualise TRITC and cell wall autofluorescence respectively. Confocal images were obtained using a Leica SP8 confocal laser‐scanning microscope (Leica Microsystems, Australia) equipped with a 40 × (NA = 1.1) or a 10 × (NA = 0.3) water immersion objective. Images were collected using HyD detectors and sequential scanning in two tracks, 405 nm excitation with 490-540 nm emission for vascular autofluorescence, and 557 nm excitation with 570-600 nm emission for TRITC. Image capture and post-acquisition image processing was carried out using ZEN blue for widefield images and for confocal images Leica Application Suite (LAS-X, Leica Microsystems). The final figure was produced using Adobe Photoshop.

### Experimental replication and statistical analyses

In Experiment 1, the cotyledons from ten biological replicates of each cultivar were sampled at 12 dpi (Table 1). In Experiment 2, the cotyledons from 14 biological replicates of each cultivar for each of the timepoints 9 and 12 dpi were sampled (Table 1). In Experiment 1, three biological replicates were processed for the WAC assay while 10 were processed for ddPCR. For both experiments, one cotyledon from each plant was processed for the WAC assay while the second cotyledon from each of the same plants were processed for PCR tests. In addition, for experiment 2, fungal biomass estimates at the crown and hypocotyl were determined for a full complement of cultivar replicates. 14 biological replicates for each cultivar for each of three respective timepoints (4, 7 and 12 weeks pi) were processed using WAC and PCR assays.

For each experiment, fluorescence measurements were taken from at least 3 technical replicates for WAC assays. Mean values for each cultivar and their respective standard errors were then calculated from the technical mean RFU values. Similarly, means and standard errors were calculated for the LmHypPro/BnHMG ddPCR droplet count ratio for each cultivar. The respective comparative WAC or ddPCR data was subjected to ANOVA tests using Microsoft Excel to generate P-values as a measure of statistical significance. Linear regression on cultivar mean data was again used to quantify the relationship between WAC and ddPCR results.

In addition, qPCR tests were performed on 19 selected cotyledon DNA extraction samples of which the LmHypPro/BnHMG PCR product ratios were subjected to linear regression analyses against their ddPCR LmHypPro/BnHMG droplet ratios to validate the accuracy of the ddPCR results (Additional file 1: Fig. S1A). In addition, 14 selected DNA extraction samples were subjected to a repeat ddPCR run and the 2 sets of ddPCR data were subjected to linear regression analysis to validate the results obtained from different ddPCR runs in the laboratory environment (Additional file 1: Fig. S1B).

In Experiment 3, the cotyledon data was subjected to similar statistical analysis described for cotyledons in experiments 1 and 2 (Fig. 5A). However, to examine effects of host cultivar and isolate on fungal biomass in cotyledons, we used beta regressions (Cribari-Neto F and Zeileis A [38] with the proportion of ‘fungal to host’ DNA as the response variable. Because our data contained zeros, which cannot be included in a beta regression, we transformed ratios using the equation t = (s × (n − 1) + 0.5)/n, where t is the transformed value, s is the original value and n is the size of the dataset (Douma and Weedon [39]). Pairwise comparisons were subsequently calculated on model least square means to determine specific differences among treatment combinations using the package emmeans. Beta regression and associated analyses were conducted in R (R Core Team [40]).

## Results

### Comparison of techniques to quantify pathogen biomass in the cotyledons

The aim of Experiment 1 was to compare WAC and PCR techniques for measuring the relative fungal biomass in cotyledons prior to senescence. *L. maculans* was detected in inoculated cotyledons at 12 dpi using WAC and PCR methods. Assays were able to consistently differentiate levels of pathogen biomass (isolate D3) in different cultivars (Fig. 1A and B). The susceptible Westar had the highest load, while the resistant Hyola350TT the lowest pathogen load (Fig. 1A and B). There was a highly significant correlation (R^2^ = 0.9965) between the WAC and ddPCR assays (Fig. 1C). The qPCR and ddPCR methods selected DNA samples were also highly significantly correlated (R^2^ = 0.9959; Additional file 1: Fig. S1A). Similar results were obtained for repeated ddPCR tests conducted on selected samples (R^2^ = 0.9956; Additional file 1: Fig. S1B). Hereafter, results of ddPCR are reported because they provide the same result. Compared to qPCR, ddPCR reagents are more expensive, but these costs are offset by lower labour costs, reduced variability as technical replication is not required and improved data handling.

### Quantification of pathogen load during disease progression

The aims of Experiment 2 were to assess assay repeatability under different environmental conditions and test a broader set of cultivars to determine whether the assays could quantify blackleg infection as disease progressed from cotyledon lesions, through the petiole and into the stem. Pathogen load was measured at different timepoints during the infection process between 9 dpi in the cotyledons/petioles and 12 wpi in the stems at plant maturity, using both WAC and ddPCR assays.

In the cotyledon lobes, pathogen load increased between 9 and 12 dpi for all cultivars but was greatest in the highly susceptible cultivar Westar (Fig. 3A, B). At 9 dpi, cultivars could be differentiated by ddPCR (Fig. 2A). The Westar, Bn1, ATR-Mako and ATR-Bonito LmHypPro/BnHMG droplet count ratios were 9.3 × 10^–2^, 5.3 × 10^–2^,2.93 × 10^–2^ and 1.3 × 10^–2^ with SE values of ± 2.4 × 10^–2^, 7.0 × 10^–3^, 7.2 × 10^–3^, and 3.3 × 10^–3,^ respectively. However, the relative fluorescence unit (RFU) levels in the WAC assays between the resistant Hyola350TT cultivar and the mock-inoculated controls were indistinguishable at 9 dpi due to the background fluorescence that occurs inherently in plant material (Ayliffe et al. [23]) supplanting the fluorescence from stained pathogen biomass. At 12 dpi, the ddPCR LmHypPro/BnHMG droplet count ratios had increased exponentially from the 9 dpi measurements across all cultivars except in Hyola350TT, as expected since it has a resistant reaction with isolate D2. The increase in pathogen load was greatest in the most susceptible cultivar Westar (14.7-fold), followed by Bn1 (10.53), ATR-Mako (12.4) and ATR-Bonito (6.08) (Fig. 2A and B). The 12 dpi WAC assay trends replicated those for the ddPCR at the same timepoint for differentiation between cultivars (Fig. 2C). At 12 dpi, the relative fluorescence unit (RFU) measurement for Westar was sevenfold higher than that of the mock inoculated samples while the RFU measurements of cvs. Bn1, ATR-Mako and ATR-Bonito were 3.88, 2.66 and 1.35 times higher, respectively, than that of the mock samples (Fig. 2C). Overall, the WAC assay distinguished the relative extent of cotyledon infections in different cultivars at 12 dpi while differentiation using ddPCR was possible at 9 dpi (Fig. 2A) prior to the development of visual symptoms (Fig. 2F).

At 9 and 12 dpi, petioles were separated from the cotyledon lobes and fungal biomass measured using ddPCR and WAC assays. At both timepoints, infected petiole samples were distinguishable from mock-inoculated controls by ddPCR (Fig. 2E). In contrast, WAC tests showed no distinction between inoculated or mock-inoculated samples (results not presented). The ddPCR cotyledon petiole measurements revealed relatively low but highly variable levels of fungal biomass (Fig. 2E). Microscopic observations of infected petioles consistently showed a single or few hyphal strands extending along the vasculature of the petioles in contrast to the lesion produced on the lobes in which there was prolific hyphal growth (Fig. 2G and H).

Crown and hypocotyl sections were harvested at three timepoints from plants in which *L. maculans* infections had progressed from cotyledons into the stem. At 4 wpi, cultivars could not be clearly differentiated with either the ddPCR or WAC assays at the crown or in the hypocotyl (Fig. 3A–D). By 7 wpi, pathogen load in the susceptible cultivar Westar could be differentiated from the more resistant cultivars by both assays in both types of tissue. Between 7 and 12 wpi, there was a sharp increase in the pathogen load of the susceptible cultivar Westar in both the crown and hypocotyl tissue as measured in both assays (Fig. 3A–D). This increase in the susceptible cultivar Westar was acutely evident in ddPCR of hypocotyl tissue in which fungal biomass measurements at 7 and 12 wpi are about tenfold higher than in the corresponding crown tissue immediately above (Fig. 3C and D). The WAC and ddPCR assays were highly correlated for both tissue types at 7 and 12 wpi with R^2^ values between 0.8112 and 0.9124 (Fig. 4A–C) but with an exception (Fig. 4D): the WAC vs ddPCR linear regression test on the hypocotyl data sampled at 12 wpi yielded an R^2^ value of only 0.4719.

### Differentiation of isolate aggressiveness by ddPCR

The aim of Experiment 3 was to determine whether the ddPCR assay could be used to phenotype the aggressiveness of *L. maculans* on cotyledons. The ddPCR results from cotyledons sampled 12 dpi differentiated the aggressiveness of D2, D8 and D22 *L. maculans* in the three cultivars tested (Fig. 5A). Overall, D22 was more aggressive than D8 which in turn was more aggressive than D2. For example, D22 pathogen load was approximately 3–4 times higher than D2 in cultivars ATR-Mako and ATR-Bonito (P < 0.0001) whereas in cv. ATR-Bonito, D2 and D8 infected cotyledons were indistinguishable. The size of the lesions on cotyledons are consistent with the respective ddPCR measurements (Fig. 5B).

## Discussion

### Comparison of techniques

This study has shown that qPCR, ddPCR and WAC techniques to quantify *L. maculans* fungal load in inoculated *B. napus* plants are well correlated. ddPCR and WAC assay results were repeatable across experiments conducted in different glasshouses and at different times of the year. In addition, the results were repeatable when applying different host-isolate treatments. The advantage of these methods is that they provide a degree of scientific rigour (repeatability, accuracy and objectivity) that cannot be achieved by traditional scoring methods. Furthermore, these methods have the potential to reduce the time required for screening as ddPCR can be implemented prior to the development of visual symptoms, or alternatively, during biotrophic phases of *L. maculans* growth such as in the petiole. Freeze-dried samples can be stored for long periods before they are processed and can be readily transported. These techniques could be applied to various aspects of the *L. maculans-B. napus* interaction. For example, these methods could be used to identify resistance in host germplasm or particularly, quantitative resistance which requires more precise phenotyping strategies. *L. maculans* isolates can be assessed for aggressiveness using these techniques or to advance our understanding of host-isolate interactions throughout disease development.

This is the first report on the use of WAC assays to measure relative fungal biomass of blackleg disease in canola. WAC assays are relatively simple, providing a directly proportional relationship between the fungal biomass and fluorescence (Ayliffe et al. [23]). The method is less costly (< A$2.00 per sample for consumables) than the PCR methods used to assess pathogen load and is amenable to processing a relatively high number of samples. Soft cotyledon and petiole samples were amenable to high-throughput processing techniques for grinding samples in 96-well plates. However, the process of individually grinding stem material and then transfer to plates was laborious. The ground tissue from more mature stem samples was not entirely amenable to suspension in solution due to the hard and fibrous nature of the material, particularly of the outer epidermal and cortical layers. This means that results from stem assays possibly reflects more the colonisation of the stem pith rather than the stem sample in its entirety. A high-throughput method has not been developed for measuring optical density when standardising the amount of material being tested for levels of chitin, which currently limits the utility of this method for economically screening large numbers of samples. It is possible that at a high throughput method could be developed. Another disadvantage of the WAC is that these tests do not discriminate between fungal pathogens or saprophytes so inter-species mixed infections or additional saprophytic infestations cannot be accounted for as is the case for the PCR assays. Moreover, canola plant material treated in the WAC method has a degree of background fluorescence that reduces the sensitivity of these measurements as was the case for wheat, grapevine, and Arabidopsis leaf material (Ayliffe, et al*.* [23]). Variation due to differential inoculation can be largely circumvented by replication of treatments and possibly pooling of replicated material (Ayliffe, et al. [23]).

The PCR techniques proved to be precise, accurate and specific in all host tissues. As far as we are aware, this is the first report on the use of ddPCR to measure relative fungal biomass in the *L. maculans-B. napus* interaction. Previously, qPCR has been used to quantify *L. maculans* fungal biomass using primers specific to the internal transcribed spacer (ITS) region (Huang et al. [30], [17] and [31]; Liu et al. [32]; Voigt et al. [33]). However, the ITS region is problematic as the copy number differs considerably between individual isolates of *L. maculans* (A. Van de Wouw, personal communication). A major advantage of the PCR methods is that the technology exists for high-throughput processing of multiple samples which includes grinding of soft tissues (leaves and petioles) and DNA extraction prior to running the PCR assays. Like the WAC assay, grinding and transfer of each individual stem sample was laborious. Consumables are more costly for both qPCR and ddPCR than chitin assays, estimated at approximately A$7.00 and A$15.00 per sample, respectively. Methods for qPCR are more laborious than ddPCR due to the requirement of technical replication to obtain statistically accurate results. On the other hand, ddPCR does not require technical replication due to the high droplet count inherent in this method. In addition, it is accepted that ddPCR is a much more sensitive measure than that of qPCR where the variation in ratio tends to be a lot less variable due to the high droplet counts of up to 20 000 per PCR reaction tube (Hindson et al. [41]; BIORAD Droplet Digital™ PCR Applications Guide [22]).

### Quantification of pathogen load in host tissues during disease progression

The assays were able to quantify pathogen load in cotyledon, petiole and stem tissues throughout the infection process, from early stages of leaf infection in seedlings to crown canker in mature plants.

For cotyledon lobes, ddPCR LmHypPro/BnHMG droplet ratio differences between cultivars were detectable at 9 dpi, prior to visual differentiation of lesions, and were consistent with results from 12 dpi as visual symptoms progressed. Visual assessment of cotyledon lesions after inoculation with a differential set of isolates is used to phenotype reactions for major gene resistance (Van de Wouw et al. [42]) and smaller leaf lesions have been correlated with presence of quantitative resistance (Huang et al. [31]). However, visual scoring of lesions on cotyledons or leaves may not provide an accurate measure of pathogen load. In this and other studies, microscopic images of cotyledon colonisation show abundant hyphae invading tissue beyond the visual boundary of the lesion (Huang et al. [31], Hubbard et al. [43]). In turn, hyphal colonisation can be significantly smaller than the areas stained for ROS (Reaction Oxygen Species involved in Programmed Cell Death) such as hydrogen peroxide using 3,3-diaminobenzidine (DAB) (Hubbard et al. [43]). The described assays could be used in conjunction with standard visual scoring techniques to provide more precise differentiation of host responses, particularly in the identification of novel sources of genetic resistance that act in the leaf during infection. The pathogen load at the cotyledon stage across all experiments were consistent resulting in low standard errors compared to other tissue types.

The presence of *L. maculans* in petioles was undetectable with the WAC assay but was detected by ddPCR. Detection of single fungal hyphae within, or multiple hyphae running along, the vasculature of the cotyledon petioles microscopically was possible using fluorescence (Fig. 2G and H) in this study. Consequently, the ddPCR cotyledon petiole measurements had very low counts and a high degree of variability with resulting high standard errors, such that the presence of *L. maculans* could be confirmed but differentiation of lines was not possible. This is consistent with Huang et al. [30], in which there was no difference in either distance grown by *L. maculans* along leaf petioles or fungal biomass measured by qPCR in petioles between cv. Darmor with quantitative resistance and the susceptible cv*.* Eurol in controlled environment experiments. In contrast, in a subsequent study by Huang et al. [17], differences were detected in growth rate and fungal biomass in leaf petioles inoculated in controlled environment experiments between two doubled haploid host lines differing in level of quantitative resistance selected from a cross between cvs. Darmor (with quantitative resistance) and Eurol (susceptible). The same wild type and GFP expressing isolates (ME24 and ME24/3.13, respectively) were used in these two studies but the authors provide no explanation for the contrasting results. While experimental conditions (18 °C and 20 °C) and timing of tissue harvest post-inoculation differed slightly between experiments, this seems an insufficient explanation if indeed this is a reliable phenotype for the identification of quantitative resistance. Although few cultivars were included in this study, the high level of variability in the petiole measurements and inability to differentiate susceptible and resistant cultivars indicates that differentiation of *L. maculans* growth in the petiole through the biotrophic phase to identify quantitative resistance may not be possible without a significant increase in replication and the pooling of petiole samples. However, further testing with a wider range of germplasm may be warranted. Screening during early seedling growth would provide a potentially useful tool for more rapid screening of resistance as opposed to waiting for plants to mature.

Using pathogen load as a proxy for resistance assumes that there is a correlation between pathogen multiplication, symptom severity and yield loss to disease (Pagán and García-Arenal [44]). In Experiment 2, *L. maculans* followed the natural disease progression pathway from infected cotyledons, through the petiole and into the stem. *L. maculans* was detected in stem samples at all timepoints from 4 wpi to maturity at both the crown where the inoculated cotyledons were attached and below in the hypocotyl, with disease progression similar in both. The disease progressed between T3 and the final measurements taken at T5 maturity, especially between T4 and T5 in the susceptible cv. Westar. The rapid increase in pathogen load is consistent with previous reports that the growth rate of *L. maculans* in the stem increases from the onset of flowering (Sprague et al. [14, 15]) and that visual symptoms of crown canker begin to appear around the onset of flowering, increasing in severity through to plant maturity (Sprague et al. [45]). In this study, significant disease progression was limited except in the susceptible cv*.* Westar. This may be because the level of quantitative resistance in the Australian commercial cultivars used in this study effectively limited blackleg progression in the stem. Blackleg resistance has been a major focus of selection for breeding programs in Australia since the devastation of the fledgling canola industry in the early 1970’s due to blackleg (Colton and Potter [46]). However, further work is required to understand precise links between *L. maculans* growth, host damage and yield which will form the focus of future work on this system.

### Differentiation of isolate aggressiveness by quantification methods

The gene-for-gene interaction between avirulence genes in *L. maculans* and the corresponding resistance gene in *B. napus* is well-characterised (Ansan-Melayah et al. [47]). Population studies on *L. maculans* characterise isolates into groups according to the virulence profile but little consideration is given to other traits that may characterise isolates, such as aggressiveness (West et al. [7]; El Hadrami and Daayf [48]; Rouxel and Balesdent [49]). In the cotyledon inoculations in Experiment 3, differences in pathogen load were detected between the isolates, each of which was virulent towards the host lines in varying degrees depending on the cultivar. The ddPCR assays presented here could be used in conjunction with screens that consider other traits related to aggressiveness (ie. sporulation rate) to identify the most aggressive isolates that could then be used to screen for host genetic resistance.

This study has shown that the WAC and PCR methods to quantify pathogen load are accurate in that the results are generally well correlated. An exception was the lower correlation at the latter stages of the stem infection (Fig. 4D, Experiment 2, T5) in the susceptible cv. Westar. We hypothesise that this may be due to the accumulation and amplification of nuclei required to generate fruiting bodies or pycnidia at the latter stages of the asexual cycle of infection in the stems, which may be the reason for the spike in ddPCR results from stem samples with advanced blackleg symptoms. Indeed, at this timepoint, pycnidial fruiting bodies were observed on some of the Westar samples at maturity. Further testing is required to ascertain the importance of pycnidial formation on the relationship between DNA quantity and fungal biomass/pathogen load. Identifying the optimal sampling time is also important to ensure the quality of both plant and fungal DNA. In initial pilot tests, inoculated cotyledons of susceptible cultivars started to senesce and drop off by 13–14 dpi. Therefore, the latest cotyledon samples were taken at 12 dpi to ensure tests at the DNA and chitin levels compared ‘living’ tissue as opposed to measurements from tissue that are in the process of senescing. Stem samples were also taken when the plants had reached physiological maturity but were still green.

## Conclusions

These relative estimates of fungal biomass provide a non-subjective, accurate assessment of pathogen load to quantify the *L. maculans-B. napus* interaction. The advantages are that they can be used prior to the development of visual symptoms or in tissues difficult to assess visually. These techniques could be used as a method to phenotype interactions requiring a higher level of accuracy for treatment differentiation, for example identification of quantitative host resistance, or in combination with currently used methods for blackleg assessments of leaf lesions and crown cankers. The corollary of this is that the assay techniques could in turn be used to determine the aggressiveness of different *L. maculans* isolates or populations in similar controlled inoculation tests that are reported in this study. These assays are not meant to substitute for experienced pathologists who can rapidly visually assess large numbers of plants, but they provide more precise and repeatable alternatives for estimating blackleg fungal biomass.

## Supplementary Information

**Additional file 1: Fig. S1.** Linear regression tests on qPCR and repeat ddPCR results (Experiment 1).

## Data Availability

The datasets used and/or analysed during the current study are available from the corresponding author on request.
