# Supplementary material for: Comparison of non-subjective relative fungal biomass measurements to quantify the Leptosphaeria maculans—Brassica napus interaction
Source: Plant Methods. 2021 Dec 1;17:122. doi: 10.1186/s13007-021-00822-6 (PMC8638343; doi:10.1186/s13007-021-00822-6)
Supplement: Supplementary file 1 — Additional file 1: Fig. S1. Linear regression tests on qPCR and repeat ddPCR results (Experiment 1). [file 13007_2021_822_MOESM1_ESM.docx]

**Fig. S1 Linear Regression tests on qPCR and repeat ddPCR results (Experiment 1).**

**A**: Linear regression tests on 19 selected cotyledon DNA extraction samples from Experiment 1 were subjected to qPCR and repeat ddPCR tests. qPCR and ddPCR LmHypPro/BnHMG ratio results revealed a R^2^ value of 0.9959 set at a confidence level of 95%. DNA samples were extracted from at least three of each of the cultivars Westar, ATR-Stingray, ATR-Sturt, ATR-Mako and ATR-Bonito (Figures 1A and B). **B**: Repeat ddPCR tests revealed a R^2^ value of 0.9956. Repeat tests whether applying the qPCR or the ddPCR methods revealed near identical results and trends.


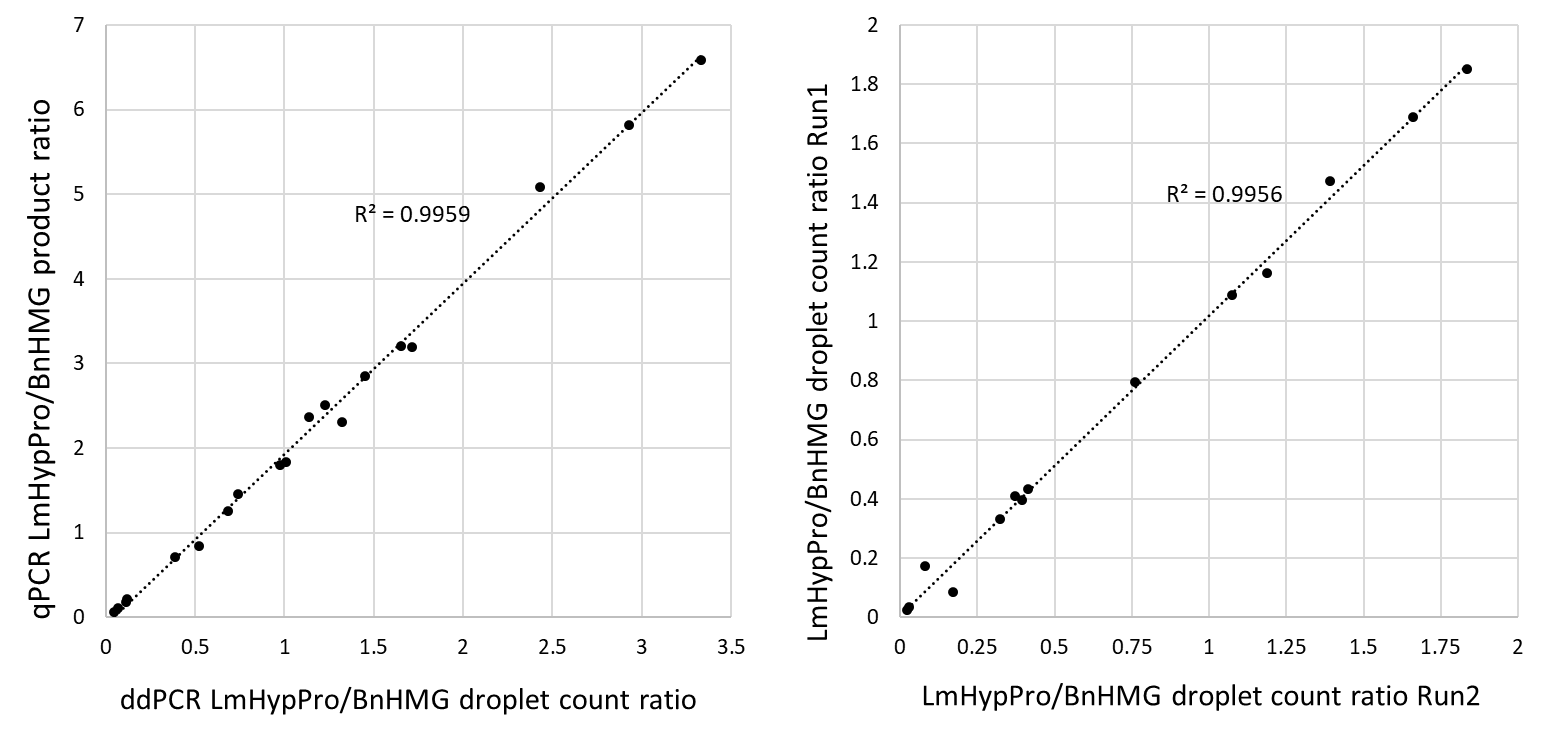


**A**

**B**
